# Spatial and Seasonal Diversity of Wild Food Plants in Home Gardens of Northeast Thailand^1^

**DOI:** 10.1007/s12231-015-9309-8

**Published:** 2015-06-20

**Authors:** Gisella S. Cruz-Garcia, Paul C. Struik

**Affiliations:** Decision and Policy Analysis Research Area, International Center for Tropical Agriculture, Km 17 Recta Cali-Palmira, Apartado Aéreo 6713, Cali, Colombia; Botanical Research Institute of Texas, 1700 University Drive, Fort Worth, Texas 76107 USA; Centre for Crop Systems Analysis, Wageningen University, P.O. Box 430, 6700 AK Wageningen, The Netherlands

**Keywords:** Abundance, frequency of occurrence, seasonality, Southeast Asia, spatial configuration, ethnobotany

## Abstract

Wild food plants (WFPs) are major components of tropical home gardens, constituting an important resource for poor farmers. The spatial and seasonal diversity of WFPs was analyzed across multi-species spatial configurations occurring within home gardens in a rice farming village in northeast Thailand. Data were collected in 77 sampling sites corresponding to five different home garden spatial configurations, namely fenced plot, fenced plot margin, yard, home garden boundary, and pot. Absolute abundance and frequency of occurrence were quantified per individual WFP species in both dry and rainy seasons, and data on additional uses (besides food) were collected through focus group discussions for each WFP species. A total of 20 species corresponding to 13 botanical families were reported. Results show that species abundance and frequency of occurrence varied seasonally and spatially within home gardens. Diversity, as observed in the analysis of Shannon and Simpson diversity indexes, also differed seasonally and across different spatial configurations. Home gardens showed higher diversity in the dry season because of the presence of human management. Ninety-five percent of the WFP species presented additional uses, with nine different types of uses in total. Finally, as this study demonstrates, the results on both the spatial and seasonal diversity of WFPs over different spatial configurations comprise a new perspective in home garden research by providing new understandings about their composition and management.

**Electronic supplementary material:**

The online version of this article (doi:10.1007/s12231-015-9309-8) contains supplementary material, which is available to authorized users.

## Introduction

Home gardens are diverse and multi-layered agro-ecosystems (Fernandes and Nair 1986) comprising small-scale production units surrounding the homestead of families maintained with family labor. Home gardens complement the functions of other farming systems such as agricultural fields. Home gardens have existed in the tropics since prehistoric times (Niñez 1985) and have probably been the oldest expression of agriculture in Southeast Asia (Wiersum 2006), playing an essential role in the process of domestication and the development of agriculture (Miller and Nair 2006). Nowadays, home gardens provide households with food, medicine, fodder, and other products grown for self-consumption and, sometimes, for sale (Galluzzi et al. 2010; Kumar and Nair 2004; Soemarwoto 1987).

Most research has focused on home gardens as integrated multi-species systems, giving greatest attention to the variation of species diversity among home gardens (e.g., Padoch and de Jong 1991; Perrault-Archambault and Coomes 2008; Peyre et al. 2006; Soemarwoto 1987). Additionally, several studies have paid special attention to the vertical variation of species by comparing the different layers of canopy strata constituting home gardens (e.g., de Clerck and Negreros-Castillo 2000; Fernandes and Nair 1986; Gajaseni and Gajaseni 1999). Nonetheless, little attention has been given to analyzing the horizontal variability within home gardens, for instance, their spatial structure and the variation of species diversity in different spatial configurations within home gardens (Abebe et al. 2010; Lok 2001; Méndez et al. 2001). Certainly, more rigorous research on the ecological basis of home gardens is needed (Nair 2001).

Home gardens possess layout structures (or configurations) that vary spatially in terms of species diversity and, in each configuration, species are mixed in specific ways, according to the particular needs of a family (Lok 2001; Méndez et al. 2001). For example, Alvarez-Buylla Roces et al. (1989) observed in southwest Mexico the presence of different areas constituting home gardens, such as the yard surrounding the house with sparsely distributed woody species, the ornamental garden densely planted mainly with herbs, the living fence, and the orchard containing useful trees and shrubs. Similarly, Greenberg (2003) observed that Mayan home gardens have different spatial configurations; for example, some plants grow along the street, others behind the house near the kitchen, and others in different kinds of containers (cans, bowls, and buckets). Home garden structure and composition have not only been studied in Latin America, but also in Africa (Abebe et al. 2010) and Southeast Asia (Wiersum 2006). Different spatial configurations within home gardens determine the presence of various habitats that will ensure the availability of useful species with diverse environmental and management requirements throughout the year.

The main function of the majority of home gardens is providing fruits and vegetables for home consumption, comprising several wild food plants (WFPs), as complements to the staples (Fernandes and Nair 1986; Hoogerbrugge and Fresco 1993; Niñez 1985). Farmers ensure the availability of food plants in their home gardens all year round (Lok 2001; Soemarwoto and Conway 1992), especially in times of stress (Cruz-Garcia and Price 2014a; Nazarea and George 1997). Additionally, the structure given to each one of the components of a home garden has an established role not only in space but also in time (de Clerck and Negreros-Castillo 2000). Vogl et al. (2004) highlighted that seasonality should be one of the main aspects that has to be taken into consideration as part of home garden research. However, little attention has been given to the study of the seasonal variation within home gardens in terms of species composition. This might partly be due to the fact that most home garden research has been carried out in the humid tropics of the developing world (Vogl et al. 2004), where there is not a well-expressed dry season.

WFPs, including fruits and vegetables consumed as complements to the staples, constitute a major component of home gardens (Chweya and Eyzaguirre 1999; Vogl-Lukasser et al. 2010). The word “wild” does not necessarily imply the absence of human management. Certainly, WFPs might be locally cultivated (*in situ* or *ex situ*), protected, tolerated, or promoted to different degrees by farmers (Casas et al. 1997; González-Insuasti and Caballero 2007; Price 1997). WFPs might be transplanted to home gardens without necessarily becoming a domesticated species, and/or could spontaneously emerge in them. Along these lines, Harlan (1975: 63) stated:

“Since domestication is an evolutionary process, there will be found all degrees of plant and animal association with man and a range of morphological differentiations from forms identical to wild races to fully domesticated races. A fully domesticated plant or animal is completely dependent upon man for survival.”

This is well illustrated in the continuum model of people and plant interactions along a gradient of management intensity illustrated by Harris (1989) and Wiersum (1997). Likewise, WFPs exist on a management continuum from “truly” wild to wild-cultivated and semi-domesticated species, excluding locally fully domesticated plants, where the management of a species might vary in time and space (Harris 1989). For instance, some cultivated species are moving toward domestication, whereas some species that used to be intensively managed are nowadays moving toward wilderness. In addition, a WFP species might be protected and promoted in some regions, but hardly ever managed in other places or even by other farmers (González-Insuasti and Caballero 2007; Ogle et al. 2001). From an ethnobotanical perspective, using an *emic* approach (based on people’s own interpretations of the environment), WFPs are species classified as “wild” and “edible” by local people, where “wild” is a cultural domain defined according to a local cognitive system (Brosius et al. 1986).

It has been widely reported that WFPs are crucial for assuring food security and dietary diversity of farming households (Akrofi et al. 2010; Cruz-Garcia and Ertug 2014; Heywood 1999; Niñez 1985). They play an important role in human nutrition, constituting an essential source of minerals, vitamins, and secondary metabolites including alkaloids, essential oils, and phenolics (Heywood 1999; Johns 2007). Plants’ edible parts range from reproductive organs like fruits, flowers, and seeds, to vegetative organs like leaves, roots, and stems (Cruz-Garcia and Ertug 2014). Nowadays, many rural societies from around the world rely on WFPs as essential components of their diet, especially during lean seasons and scarcity periods (Cruz-Garcia and Price 2014a; Etkin 1994; Heywood 1999; Turner and Davis 1993). However, the seasonal and spatial diversity of species, including WFPs, within home gardens have not received enough attention in home garden research (Chweya and Eyzaguirre 1999; Vogl-Lukasser et al. 2010). Certainly research of the different spatial and temporal scales of species diversity within home gardens is undoubtedly necessary in order to achieve a better understanding of the relation between home garden sustainability and diversity (Abebe et al. 2010; Torquebiau 1992).

This paper documents the spatial and seasonal diversity of WFPs in home gardens in a rice farming village of Kalasin, northeast Thailand, where it has been previously documented that WFPs from home gardens play an essential role in the diet of local people (Cruz-Garcia and Price 2014a; Moreno-Black et al. 1996a). The hypotheses underlying this article are that WFP species vary spatially and seasonally within home gardens, and multiple use types of WFPs occur in the different spatial configurations. In order to test the hypotheses, we (a) quantified the seasonal abundance and frequency of occurrence of individual gathered plants (climbers, herbs, shrubs, and trees) in different spatial configurations within home gardens, (b) compared different spatial configurations in terms of their diversity indexes, and (c) quantified the multiple additional uses of WFP species (Cruz-Garcia and Price 2011) in relation to the spatial configurations where they grow.

## Methodology

### Study Area

**Fig. 1:**
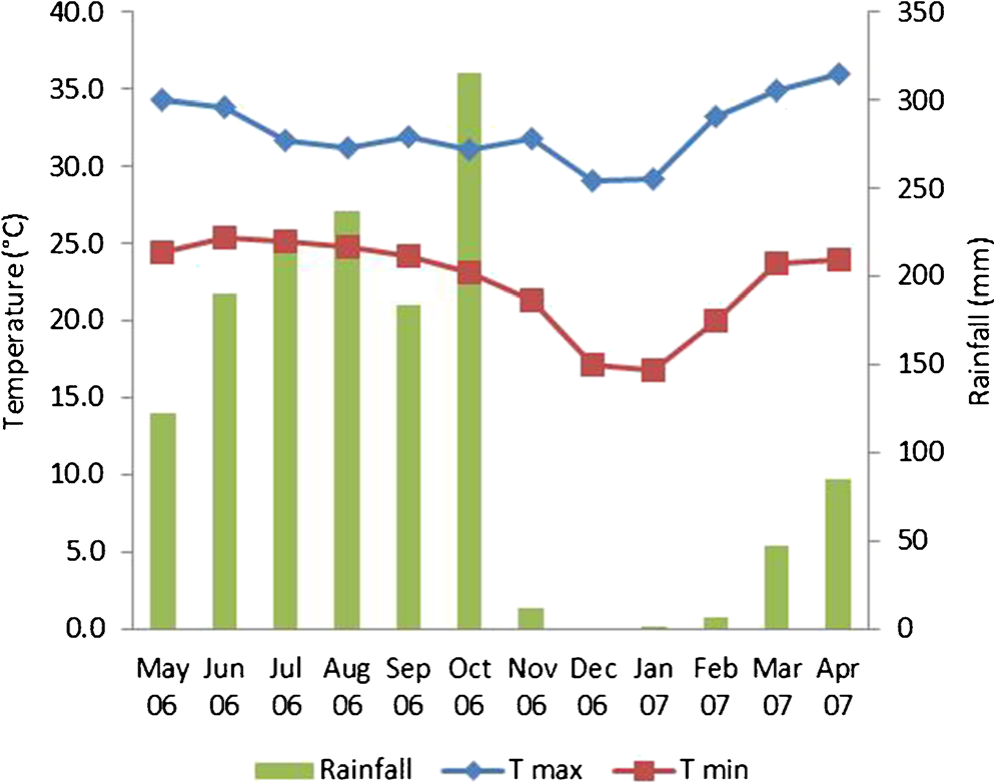
Maximum temperature (T max), minimum temperature (T min), and rainfall during the period of data collection (May 2006 until April 2007).

The study was conducted in Kalasin province, located in the northeast of Thailand, which is the poorest region in the country (National Statistical Office of Thailand 2001). The study area is characterized by having heavily leached fine sandy loam, highly saline and poorly drained soils, with low quantities of organic matter, phosphates, and nitrogen, at an elevation that ranges from 100 to 300 m asl (Parnwell 1988). Northeast Thailand has a Tropical Savannah climate, corresponding to “Aw” in the Köppen climate classification system, with a rainy season from May through October, and a dry season from November through April. The dry season includes a cool period followed by a hot period (Tomita et al. 2003; Wijnhoud 2007). Meteorological data were provided by Kamalasai station in Kalasin Province, the nearest to the research area, for the complete time range of data collection that started in May 2006 at the beginning of the rainy season 2006 and finished in April 2007 at the end of the dry season 2006–2007 (Fig. 1). The monthly average rainfall in the rainy season was 210 mm, and in the dry season it was 25 mm. The rainy season thus comprised 88% of the annual rainfall in the area. The natural vegetation of this region is dry monsoon forest, mainly composed of dry dipterocarp forest (Parnwell 1988; Prachaiyo 2000). However, the forest area has decreased from 90% in the 1930s to less than 14% in 2004, due to the extension of the agricultural area and population growth (Wijnhoud 2007).

The population of northeast Thailand, which has farming as primary occupation, has adjusted to this environmental variability by developing a combined subsistence system that depends on glutinous rice as main source of income and dietary staple, corresponding to 70% of the arable land of the region (Wijnhoud 2007). Glutinous rice is usually paired with the consumption of wild foods, including WFPs, crabs, insects, frogs, fish, and mushrooms (Cruz-Garcia and Price 2011; Moreno-Black et al. 1996a; Price 1997). Cultivation of rain-fed transplanted glutinous rice in paddy fields occurs in the rainy season, whereas in the dry season farmers cultivate either direct seeded rice, mushrooms, and vegetables, or cannot practice agriculture, depending on their access to irrigation (Cruz-Garcia and Price 2011).

Northeasteners constitute one of the largest minority groups in the country. The majority are ethnically of Lao origin. The most widely spoken language in the region is called *Isaan*, which is Lao with Thai influence, written using Thai scripts. Thai is formally learned at school. The society in northeast Thailand is characterized by having a pattern of matrilocal residence, along with a customary inheritance of land through women (Price and Ogle 2008). Given the increasing rate of outmigration and the important role that remittances are playing for the family economy, the traditional matrilocal stem family cycle is currently being affected (Prapertchob 2001).

Kalasin Province has a population of approximately one million inhabitants, with a density of 132.3 inhabitants/km^2^. Families have four members on average and 24% are female-headed households. Northeasteners (99.5%) practice Theravada Buddhism. The population has attended school for an average of 6.5 years. Fifty-two per cent of the population is constituted by unpaid family workers, and 36% are engaged in self-employment, mostly in agriculture (National Statistical Office of Thailand 2001).

**Fig. 2:**
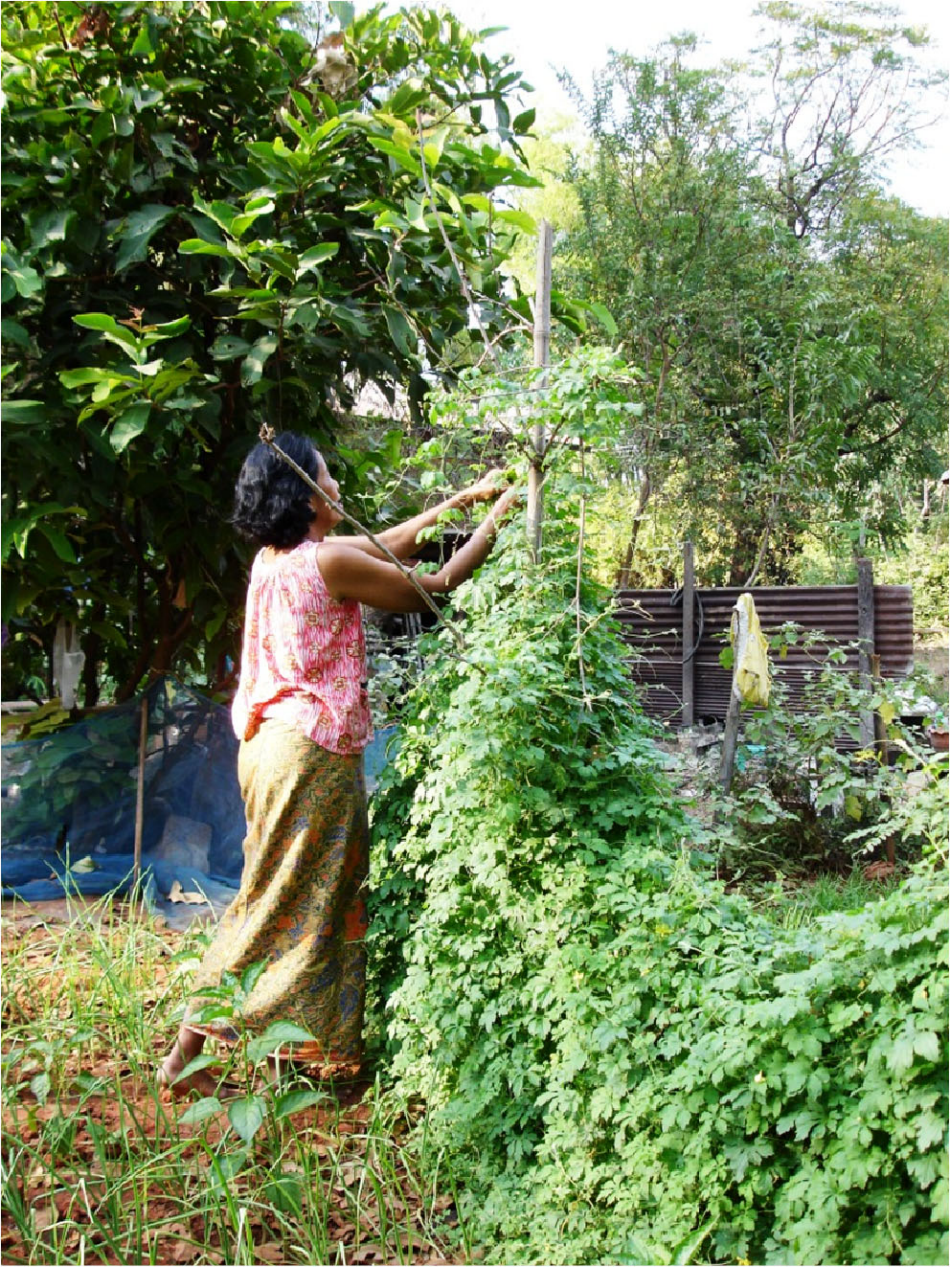
Home garden in Ban Sa-at Tai, Kalasin, northeast Thailand.

In Kalasin, WFPs are gathered from forests, fields, and home gardens, among other places (Cruz-Garcia and Price 2011; Moreno-Black and Somnasang 2000; Price 1997). Cruz-Garcia and Price (2014a) explained that WFPs play an essential role in the food security of rural families, particularly for the most vulnerable households. WFPs are an important component of home gardens (Moreno-Black et al. 1996b; Price 2005; Wester and Yongvanit 1995), where women, who inherit the land, play an essential role in their maintenance (Moreno-Black et al. 1996b; Price and Ogle 2008). In Kalasin, farmers not only gather WFPs, but also actively cultivate and manage them in their home gardens, ensuring their availability throughout the year (Cruz-Garcia and Price 2014b; Somnasang and Moreno-Black 2000). In this way, Moreno-Black et al. (1994, 1996b) reported that 29% of the useful plant species growing in home gardens in northeastern Thailand are locally classified as wild, and 95% of households presented WFPs in their home gardens (Fig. 2). The presence of WFPs in home gardens is related to their availability in other areas of the farming landscape. For instance, in research conducted in the same site where this study took place, Cruz-Garcia and Price (2014b) found that farmers actively transplant WFP species to home gardens using transplanted material not only from other home gardens and markets, but also from rice fields, upland fields, and secondary woods.

Farmers from Kalasin conceptually distinguish WFPs, which are grouped under the term “geht eng,” meaning in *Isaan* language “birth itself”. Nonetheless, they differentiate “birth itself” *type* of plant (WFP) from the *verb* “to birth by itself,” which means to grow without human intervention, i.e., without the action of transplanting or sowing. This distinction is necessary, given that some “geht eng” species (birth itself *type* of plant) can be transplanted or sown. In addition, domesticates that “birth themselves” are not considered “birth itself” *type* of plants; for instance, tomatoes that grow from consumption debris are not considered WFPs (Price 1997).

### Methods of Data Collection

The research was conducted in a rice farming village in northeast Thailand called Ban Sa-at Tai, situated in Tambon Nua, Ampher Muang, Kalasin Province (145 masl). Ban Sa-at Tai was selected for this study because previous research conducted in this village by the senior author (Cruz-Garcia and Price 2011, 2014a, 2014b) and other researchers (Moreno-Black and Somnasang 2000; Moreno-Black et al. 1996b; Price 1997) documented that WFPs constitute a major component of home gardens, where farmers keep and actively gather these species throughout the year for home consumption and, sometimes, for sale.

Twenty home gardens corresponding to 20 different households were randomly selected in the village. This constitutes 36% of all households present in the village, taking into account that all households have a home garden. Five different spatial configurations occurring within home gardens, namely fenced plot, fenced plot margin, yard, home garden boundary, and pot, were identified together with community experts. Fenced plots are small fenced areas within the home garden where farmers mainly grow domesticated plants and a few WFPs to use for food (vegetable) and medicine. Fenced plot margins include a 0.5 m wide border surrounding a fenced plot. Yards comprise the home garden area surrounding the house but excluding fenced plots and their margins, hedgerows, fences, and pots. Yards are characterized by widely spaced vegetable plants and trees. Home garden boundaries include hedgerows and fences that delimit the household compound and respective home garden, which are mainly comprised of woody species. Pots are either small containers made of ceramic with a mean diameter of 0.25 m, or big containers made of old tires with a diameter of 0.50 m. Depending on their size, pots could have only one species or mixtures of plants, and one home garden could have more than one pot. A home garden did not necessarily possess all five types of spatial configurations.

In order to analyze the spatial and seasonal variation of WFPs within home gardens and to compare spatial configurations according to species diversity, the abundance and frequency of occurrence of all WFP species growing in each one of the 20 selected home gardens were registered for each spatial configuration in both seasons. This yielded a total of 77 sampling sites, where one sampling site refers to one specific spatial configuration in a particular home garden (Table 1). Altogether, the total sampled area covered 2,749 m^2^. Sampling sites were analyzed in August 2006 (rainy season) and February 2007 (dry season). Data for the dry season were collected in February before temperatures rise and it becomes difficult to maintain WFPs in a home garden.

**Table 1:** Total of sampling sites per spatial configuration within home gardens and area covered.

Spatial configuration	Number of sampling sites	Sampled area (total in m^2^)
Fenced plot	12	182
Fenced plot margin	12	126
Yard	20	2,266
Home garden boundary	12	172
Pot	21	3

With the purpose of quantifying the occurrence of specific use types of WFP species in different spatial configurations within home gardens, data on multiple uses were registered for each one of the observed species through focus groups and complemented by interviews with local experts, as explained in Cruz-Garcia and Price (2011). Focus groups and interviews were conducted in *Isaan* language with the help of local translators. Focus groups were carried out with six to nine middle-aged or slightly older women (34 to 66 years old) that were identified as knowledgeable on WFPs by the villagers. Informants were women, given that it has been reported for this village that women are the knowledge holders on WFP use and management (Moreno-Black et al. 1994; Price 2003; Price and Ogle 2008). All informants who participated in this study did so freely and with consent.

Data collection was carried out with the authorization of the National Research Council of Thailand (NRCT) and in adherence to the International Society of Ethnobiology Code of Ethics (2006).

### Methods of Data Analysis

The botanical names of the species were based on research conducted in the same village by the senior author, who published a list of 87 WFPs (Cruz-Garcia and Price 2011). In this study, plant names were obtained by elicitation in focus groups conducted with local informants who were asked to list the plants they regard as “wild” and “edible.” The local names of the plants were recorded in the local Thai-Lao vernacular names using Thai script, and their botanical identification was carried out by taxonomists from the Department of Biology of Chang Mai University and Walai Rukhavej Botanical Research Institute of Mahasarakham University. Herbarium specimens of most of identified species are on repository in one or more locations in Thailand, including the Herbarium of Walai Rukhavej Botanical Research Institute (WRBG) in Mahasarakham, the Bangkok Herbarium of the Department of Agriculture (BK) in Bangkok, and the Herbarium of Khon Kaen University (KKU) in Khon Kaen. Botanical naming of species, genus, and family follows “Flora of Thailand” (Bangkok Forest Herbarium).

Data were analyzed per species, spatial configuration, and season. Absolute abundance and frequency of occurrence were calculated per WFP species for each spatial configuration and the whole home garden ecosystem in both seasons (dry and rainy). Absolute abundance, referred to as Ab, is the number of individual plants of a species per unit area (100 m^2^, otherwise indicated), estimated by the sum of the number of individual plants divided by the total area (m^2^) of all sampling sites belonging to a spatial configuration. Frequency of occurrence (presence frequency) is the percentage of observations where the species was present: (a) the percentage of sampling sites where the species occurred in a spatial configuration, referred to as Freq_SS_, and (b) the percentage of spatial configurations where the species occurred, referred to as Freq_SUB-S_. These measurements (Ab, Freq_SS_, and Freq_SUB-S_) complement each other.

Species richness and Simpson and Shannon diversity indexes were calculated per spatial configuration. Species richness (Sp_d_) was obtained as species density, quantifying the number of species per unit area (100 m^2^, otherwise indicated). The Shannon and the Simpson indexes, referred to as H’ and D respectively, were obtained according to Magurran (2004).

Finally, the amount and variety of multiple uses of WFPs present in each spatial configuration within home gardens were calculated.

## Results

### Home Gardens and Their Spatial Configuration

All sampled home gardens (n = 20) presented WFPs in at least one season and 90% in both seasons, with a mean of 4.1 species per home garden in the dry season (SD = 2.8) and 3.6 in the rainy season (SD = 3.0). However, home gardens differed in the number and type of spatial configurations they contained. Home gardens showed a mean of three different spatial configurations (SD = 1.7) in a mean area of 137 m^2^ (SD = 184 m^2^). Thirty percent of home gardens showed all five spatial configurations, and 10% presented four spatial configurations, whereas 15% showed three spatial configurations, 12% two spatial configurations, and 30% only one spatial configuration. Home gardens with a bigger area usually possessed more spatial configurations. For instance, home gardens with four or five spatial configurations had a mean area of 231 m^2^ (SD = 150 m^2^), and the mean area of home gardens with one or two spatial configurations was 84 m^2^ (SD = 220 m^2^). All spatial configurations, except yards, were observed in 50% of home gardens. Yards were present in 95% of all sampled home gardens.

Management varied per type of spatial configuration within home gardens. WFPs growing inside fenced plots were generally transplanted, watered, and protected. The plot fence also prevented the entrance of animals into the garden. In fenced plot margins, plants were not protected against animals, and farmers usually did not take care of the plants growing in this area directly (as they did for these plants growing inside the fence), but they did it rather indirectly; for instance, these plants incidentally received water and nutrients applied to the species growing inside the fence. Plants growing in yards might be transplanted and/or protected, for example by placing sticks around small trees. In addition, some species that were important for the local cuisine were planted near the kitchen, water jar, or toilet so they could indirectly receive water while cooking and washing the dishes or body. Plants growing in home garden boundaries had mainly been transplanted and were pruned. Plants in pots had been transplanted, were protected, and, when necessary, were watered. Pots were placed on the top of columns or walls higher than one meter, so chickens and other animals could not destroy them.

### Seasonal Abundance and Frequency of Occurrence of Wild Food Plants (WFPs) in Different Spatial Configurations

A total of 20 WFP species corresponding to 13 botanical families were observed, and 1,390 individuals were counted, with a mean of 0.5 individuals per m^2^. The family with the highest number of species was Leguminosae (six species), followed by Cucurbitaceae and Menispermaceae (two species each). All species were observed in both seasons, except for the weedy herb *Limnophila aromatica* Merr. that was only observed in the rainy season (Appendices 1, 2, 3, and 4, Electronic Supplementary Material [ESM]). According to their growth form, 45% of species were trees, 20% climbers, 20% terrestrial herbs, 10% shrubs, and one species was a rattan. Only three species were annual (one climber and two herbs), whereas the rest were perennials.

The tree *Tamarindus indica* L. was the most abundant species in both dry and rainy seasons, with 286 and 386 individuals counted respectively (a hundred more individuals were counted in the rainy season because new seedlings started to grow due to the presence of rainfall). Tamarind, which mainly grows in home garden fences, yards, and fenced plots, is an important tree in the region with multiple uses as food. For instance, its fruit, locally called “bak kaam,” is widely consumed ripe and unripe as snack, or its juice is added to some dishes, and its shoots are eaten as vegetable.

In the dry season, the perennial herb *Centella asiatica* (L.) Urb. also showed a high absolute abundance (284 individuals observed in the total sampled area). *C. asiatica* is a medicinal herb very common inside fenced plots, where it showed its highest density (137 individuals per 100 m^2^). This species was also present in yards and pots. The tree *Leucaena leucocephala* (Lam.) de Wit was also abundant in this season. *L. leucocephala* has multiple edible parts (shoots, leaves, and fruits) and many additional uses besides food such as medicine, fuel, and fodder. The annual herb *Amaranthus viridis* L., the tree *Phyllanthus acidus* (L.) Skeels, the rattan *Calamus* sp., and the perennial climber *Tiliacora triandra* Diels were also abundant in this season.

In the rainy season, the annual herb *L. aromatica* and the tree *L. leucocephala* were also abundant, with 79 and 54 individuals counted, respectively. *L. aromatica* or “phak kayeng” is commonly transplanted from rice fields, where it naturally grows, to home garden pots (2,633 individuals per 100 m^2^). This plant, which is also used as medicine, constitutes an important ingredient of the local cuisine, especially liked for its aromatic smell. *A. viridis*, *C. asiatica*, *P. acidus,* and the perennial herb *Ipomoea aquatica* Forssk. were also abundant during this season.

The species with the highest frequency of occurrence in spatial configurations within home gardens (Freq_SUB-S_) were *T. triandra* and the tree *Spondias pinnata* Kurz, both found in 80% of the spatial configurations in the dry and rainy season. *T. triandra*, locally called “yaa nang,” plays an essential role in local cuisine and as medicine, reasons why most households transplant it to their home gardens. The fruit of *S. pinnata*, called “bak kawek,” characterizes the “som tam” (papaya salad) prepared and frequently consumed in this region, in contrast with the “som tam” prepared in the rest of the country.

In the dry season, 47% of the plants were observed in three or more spatial configurations, whereas in the rainy season the majority of plants (85%) were only found in one or two spatial configurations. WFPs presented low abundance (Ab) and frequency of occurrence (Freq_SS_) among sampling sites of fenced plots. The only species observed in more than one home garden’s fenced plot were *T. indica* and the perennial climber *Coccinia grandis* (L.) Voigt in the dry season, and the perennial climber *Cissampelos pareira* L. in the rainy season. WFPs also presented low abundance (Ab) and frequency of occurrence (Freq_SS_) in fenced plot margins, with no species, except for *A. viridis*, observed consecutively in both seasons. Yards showed considerably more WFP species than other spatial configurations, with *T. indica* as the most abundant plant occurring in 50% of the sampling sites (Freq_SS_). In home garden boundaries, which include hedgerows and fences, the most abundant plants were *T. indica* and *L. leucocephala* (Freq_SS_ = 50%), which grow as shrubby trees in this spatial configuration due to pruning. In pots, the most abundant plants were *C. asiatica* in the dry season and *L. aromatica* in the rainy season.

### Spatial and Seasonal Variation of Species Diversity within Home Gardens

The diversity of WFPs in home gardens was notably greater in the dry season, not only with regard to the number of individuals observed in the total sampled area (771 and 619 individuals in dry and rainy seasons, respectively), but also the mean number of individuals and species across all sampled home gardens (Table 2). Moreover, the means of home garden species density (Sp_d_), Shannon (H’) index, and Simpson (D) index indicated that WFP diversity was higher in the dry season. Number of individuals, number of species, and all diversity indexes, however, presented great variability across home gardens in both seasons, which was reflected in their high standard deviations.

**Table 2:** Mean and standard deviation of number of individuals, number of species, species density (Sp_d_), Shannon diversity index (H’), and Simpson diversity index (D) of WFPs across all sampled home gardens in both dry and rainy season.

	Dry season		Rainy season	
	Mean	SD	Mean	SD
Number of individuals	39	113	31	83
Number of species	4.1	2.8	3.6	3.0
Sp_d_^1^	12.0	21.7	7.3	6.6
H'	1.06	0.51	0.81	0.66
D	0.29	0.31	0.36	0.36

^1^ Number of species per 100 m^2^.

The results showed that 60% of spatial configurations (yards, home garden boundaries, and pots) presented higher species density and greater diversity in the dry season, whereas 40% (fenced plots and their margins) did so in the rainy season (Tables 3 and 4). Yards presented the highest diversity but lowest Sp_d_ in both seasons because they have much bigger areas within home gardens where WFPs are heterogeneously spread, with no visibly dominant species. Fenced plots showed the highest species density in the dry season, but showed the lowest diversity (H’, D), which can be partly explained by the dominance of *C. asiatica*. In the rainy season, fenced plots, where farmers cultivate different species of domesticated, wild vegetables and other herbs taking advantage of the rainfall, and fence garden margins showed the highest diversity after yards. Pots and home garden boundaries showed the lowest diversity in this season due to the dominance of *L. aromatica* in pots, and *T. indica* and *L. leucocephala* as major constituents of living fences.

**Table 3:** Species density (Sp_d_), Shannon diversity index (H’), and Simpson diversity index (D) of WFPs per spatial configuration in the dry season.

Spatial configuration	Sp_d_^1^	H'	D
Fenced plot	5.49	0.28	0.91
Fenced plot margin	2.38	0.74	0.54
Yard	0.79	2.58	0.09
Home garden boundary	4.65	0.74	0.59
Pot	N.A.^2^	0.54	0.72

^1^ Number of species per 100 m^2^.

^2^ Not applicable because of the small sampled area.

**Table 4:** Species density (Sp_d_), Shannon diversity index (H’), and Simpson diversity index (D) of WFPs per spatial configuration in the rainy season.

Spatial configuration	Sp_d_^1^	H'	D
Fenced plot	2.75	1.43	0.19
Fenced plot margin	3.97	1.50	0.16
Yard	0.66	2.36	0.11
Home garden boundary	2.91	0.45	0.76
Pot	N.A.^2^	0.46	0.74

^1^ Number of species per 100 m^2^.

^2^ Not applicable because of the small sampled area.

### Multiple Additional Uses of WFP Species in Relation to the Spatial Configurations Where They Grow

All WFP species, except for *Barringtonia acutangula* (L.) Gaertn., have additional uses besides food, constituting altogether up to nine different types of uses. For example, tamarind, besides food, has six additional uses (medicine, timber, fuel, fodder, dye, and cleaning) and provides a place of shadow outside the house within the household compound. The types of uses covered by the WFPs listed in the present study were: medicinal (95% of species), animal fodder (25%), fuel (20%), domestic (15%), timber (10%), dye (10%), cleaning (10%), auxiliary (5%), and ritual (5%).

**Fig. 3:**
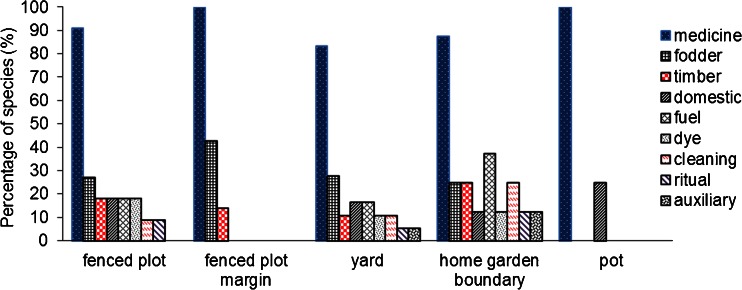
Percentage of wild plant species presenting multiple uses besides food per spatial configuration within home gardens.

Yards and home garden boundaries comprised WFP species belonging to the nine different types of use additional to food, whereas those species growing in pots belong altogether only to two use types. The percentage of species belonging to each use type, except for medicine (which was present in all spatial configurations), varied per spatial configuration (Fig. 3).

## Discussion

### Spatial Variation of WFP Species within Home Gardens

The results of this study revealed that WFP species vary spatially within home gardens, which is reflected in the following findings: (a) home gardens comprise a wide array of structurally different spatial configurations with multiple species assemblages; (b) diversity, as observed in the analysis of Sp_d_, H’ and D, was different in each spatial configuration; and (c) the seasonal abundance and frequency of occurrence of WFP species varies when comparing different spatial configurations within home gardens. The large variability of the spatial structure and diversity in home gardens has also been reported in other countries, for example, in southern Ethiopia (Abebe et al. 2010), southeast Mexico (Alvarez-Buylla Roces et al. 1989), central Sulawesi in Indonesia (Kehlenbeck and Maass 2004), and central Vietnam (Vlkova et al. 2011). These studies, however, did not present a detailed comparison of WFP species across different spatial configurations.

The research findings revealed that the spatial structure in terms of number and types of spatial configurations within home gardens is diverse and varies from household to household. Certainly, home gardens integrate annual and perennial species of trees, shrubs, and herbs, often in combination with livestock, recreating diverse habitats important for plants and animals (Fernandes and Nair 1986; Kehlenbeck et al. 2007; Torquebiau 1992). The species composition, size, and location of each structural assemble of home gardens are defined by local management strategies (Kumar and Nair 2004). Indeed, the results of this study showed that types of human management differ across spatial configurations within home gardens, a phenomenon also explained by Lok (2001).

Many plant species have specific niche requirements, which is reflected in the fact that 85% of the species in the rainy season and 53% in the dry season were found in only one or two spatial configurations in the study site. Although yards were the most common and diverse, all spatial configurations should be regarded as equally important for the maintenance of WFP diversity, given that they provide different habitats for plant species, complementing each other spatially and seasonally. In this way, each structural ensemble of home gardens conforms to a specific niche, which is intrinsically related to the others (Kumar and Nair 2004). Certainly, the diversity of plant species present in home gardens has been acknowledged as an important factor for their sustainability and productivity (Kehlenbeck et al. 2007).

### Seasonal Variation of WFP Species within Home Gardens

The research results illustrate that WFP species vary seasonally within home gardens, which is echoed in the following findings: (a) the number of individuals, number of species, and species diversity (Sp_d_, H’, and D) varied in dry and rainy seasons across all sampled home gardens; (b) diversity, as observed in the analysis of Sp_d_, H’, and D, differed per season across different spatial configurations within home gardens; and (c) WFP species abundance and frequency of occurrence varied seasonally when comparing spatial configurations within home gardens. This quantitatively shows that spatial configurations can vary temporarily or cyclically, as previously stated by Lok (2001). In addition, WFP diversity in home gardens greatly differs across households in both seasons, as reflected in the high standard deviations for all diversity indexes, number of individuals, and number of species. Certainly, Kehlenbeck et al. (2007:304) emphasized that “no individual factor alone determines the plant diversity found in home gardens, but rather a complex combination of agro-ecological, socio-economic, cultural, and political factors causes spatial and temporal variation of plant species.”

The differences observed between both dry and rainy seasons were substantial. In the dry season, home gardens showed (a) higher mean number of individuals and number of species across households, (b) higher WFP diversity according to Sp_d_, H’, and D indexes, and (c) higher diversity of WFPs in more than half of the spatial configurations. These findings are contrary to the initial expectation that the rainy season would present a higher diversity given that the presence of rainfall facilitates the growth of most species. Therefore these results cannot be explained by physical environmental factors alone, because human management is certainly a major factor for assuring the maintenance of WFP diversity under the presence of higher environmental stress. For instance, other studies conducted in the same village emphasized that WFPs are not only tolerated when they grew spontaneously, but also actively transplanted and protected (Cruz-Garcia and Price 2014b; Moreno-Black et al. 1996b). For instance, the senior author found that 98% of households transplanted WFP species into home gardens (Cruz-Garcia and Price 2014b). In addition, WFPs growing in home gardens are particularly important for local families when their availability decreases in other ecosystems of the farming landscape (e.g., rice fields), which certainly occurs during the lean months corresponding to the dry season (Cruz-Garcia and Price 2014a). Indeed, it has also been emphasised for other countries that farmers ensure the availability of food plants in their home gardens throughout the year, especially in times of stress (Lok 2001; Nazarea and George 1997; Soemarwoto and Conway 1992). Management of WFPs in home gardens has also been reported in other places in the world, for example in Eastern Tyrol (Vogl-Lukasser et al. 2010) and Bangladesh (Millat-e-Mustafa et al. 2000).

### The Specific Roles of WFPs

This study underlines the importance of WFPs as components of home gardens, reflected in the fact that 90% of home gardens presented WFPs in both seasons, with the quantification of 20 species. Indeed, previous research conducted by the senior author in the same study site highlighted that all sampled households (n = 40) gathered WFPs from home gardens for home consumption (Cruz-Garcia and Price 2014a). This is clearly aligned with Chweya and Eyzaguirre (1999), who emphasized that WFPs are an important constituent of home gardens and household food security.

Research results reinforce the statement that domestication is a locally differentiated concept and process, given that a species can be simultaneously managed differently at various places (Cruz-Garcia and Price 2014b; González-Insuasti and Caballero 2007). Therefore some of the species that are locally regarded as “wild” in the study site could be classified as “domesticated” in other regions. In addition, the ethnobotanical approach to domestication, which is based on the characterization of WFPs as a cultural domain according to local cognitive systems, also allows that a species that is regarded as “wild” in one location might be classified as domesticated in another place or by scientists. For instance, this is the case for *Cajanus cajan, Tamarindus indica*, and *Psidium guajava,* which are considered “wild” by farmers in Ban Sa-at Tai village in Kalasin but classified as domesticated species by scientific literature. Certainly, it has been argued that local categories of “wild” do not necessarily match scientific or non-local categories of “wild” (Michon and De Foresta 1997).

Along these lines, it is also important to highlight that many WFPs have been scientifically classified as weeds in the scientific literature (HEAR 2007). Small-scale farmers, however, tolerate, encourage, and/or protect in their home gardens a variety of weeds (that usually are not classified as “weeds” from an *emic* perspective) given the multiple uses they have (e.g., Datta and Banerjee 1978; Kim et al. 2007; Mukhopadhyay 1995; Van Chin 1999; Vogl-Lukasser et al. 2010; Vongsaroj and Nuntasomsaran 1999). Several weed species are edible, and the consumption of weeds has been reported around the world (e.g., Cruz-Garcia and Price 2012; Díaz-Betancourt et al. 1999; Duke 1992; Grivetti et al. 1987; Rapoport et al. 1995; Sinha and Lakra 2007; Vogl-Lukasser et al. 2010). Certainly, it has been documented for the study site that, despite the fact that 66% of the locally consumed wild vegetables are regarded as weeds by the scientific literature, the highest CSI (Sutrop’s Cognitive Salience Index) scores of all wild vegetables free-listed by local informants (village census) corresponded to weeds, which indicates the cultural cognitive importance that these plants have for local households (Cruz-Garcia and Price 2012). Indeed, 70% of the WFPs observed in home gardens were reported as weedy vegetables in the previous study, and five WFP species found in home gardens (*I. aquatica*, *L. aromatica*, *C. asiatica*, *C. grandis,* and *L. leucocephala*) were among those wild vegetables with the highest cultural cognitive importance.

The findings of this study showed that multiple use types of WFPs occur in the different spatial configurations within home gardens, where WFPs presented up to nine additional uses besides food. Indeed, it has been reported that home gardens around the world are characterized by the presence of multiple purpose species (Fernandes and Nair 1986; Galluzzi et al. 2010; Méndez et al. 2001), given that an important factor for selecting the species to grow in home gardens is their variety of uses and derived products (Gajaseni and Gajaseni 1999). The results of this study also illustrated the presence of species with multiple uses across spatial configurations, indicating that each spatial configuration in a home garden has multiple use types of WFPs that vary per season with the change in species composition.

Remarkably, almost all WFPs (95% of species) are also locally utilized as medicine in the study site. This food/medicine overlap has also been documented in other places as a major characteristic of WFPs (Etkin and Ross 1982; Ogle et al. 2003; Pieroni and Quave 2005; Vandebroek and Sanca 2007).

## Conclusions

The research findings provide strong evidence to conclude that WFP species vary spatially and seasonally within home gardens, and multiple use types of WFPs occur in the different spatial configurations that are comprised within home gardens. In this way, home gardens offer a wide array of structurally different habitats presenting different species assemblages that allow the presence of a great diversity of useful and multipurpose species during the whole year. Finally, as this study demonstrates, the results on both the spatial and seasonal diversity of WFPs over different spatial configurations feature a new perspective in home garden research by providing new understandings about their composition and management.

## Electronic supplementary material

ESM 1 (DOCX 34 kb)
