# Supplementary material for: Spatial and Seasonal Diversity of Wild Food Plants in Home Gardens of Northeast Thailand1
Source: Econ Bot. 2015 Jun 20;69(2):99–113. doi: 10.1007/s12231-015-9309-8 (PMC4490187; doi:10.1007/s12231-015-9309-8)
Supplement: Supplementary file 1 — (DOCX 34 kb) [file 12231_2015_9309_MOESM1_ESM.docx]

**<H1>Appendices**

**Appendix 1.** Wild food plants observed in the dry season, classified by growth form and ordered by family and scientific name, indicating absolute abundance (Ab) as number of individuals of a species per 100 m^2^.

| Botanical family | Scientific name | Fenced plot | Fenced plot margin | Yard | Home garden boundary | Pot |
| --- | --- | --- | --- | --- | --- | --- |
| Climber |  |  |  |  |  |  |
| Cucurbitaceae | *Coccinia grandis* (L.) Voigt | 2.0 | 0.8 | 0.2 |  |  |
|  | *Momordica charantia* L. |  |  | 0.2 | 0.6 | 66.7 |
| Menispermaceae | *Cissampelos pareira* L. | 0.5 | 0.8 | 0.1 |  |  |
|  | *Tiliacora triandra* Diels | 0.5 |  | 0.3 | 2.3 | 1.0 |
| Rattan |  |  |  |  |  |  |
| Arecaceae | *Calamus* sp. |  |  | 0.6 |  |  |
| Shrub |  |  |  |  |  |  |
| Leguminosae | *Cajanus cajan* (L.) Millsp. |  |  | 0.4 |  |  |
|  | *Senna sophera* (L.) Roxb. |  |  | 0.2 |  |  |
| Terrestrial herb |  |  |  |  |  |  |
| Amaranthaceae | *Amaranthus viridis* L. | 2.0 | 4.8 | 0.6 |  |  |
| Convolvulaceae | *Ipomoea aquatica* Forssk. |  |  | 0.3 |  |  |
| Umbelliferae | *Centella asiatica* (L.) Urb. | 137.4 |  | 0.4 |  | 9.0 |
| Tree |  |  |  |  |  |  |
| Anacardiaceae | *Spondias pinnata* Kurz | 0.5 |  | 0.4 | 0.6 |  |
| Clusiaceae | *Cratoxylum formosum* (Jack) Benth. & Hook.f. ex Dyer | 0.5 |  | 0.9 |  |  |
| Euphorbiaceae | *Phyllanthus acidus* (L.) Skeels | 0.5 |  | 0.6 | 1.7 |  |
| Lecythidaceae | *Barringtonia acutangula* (L.) Gaertn. |  |  | 0.1 |  |  |
| Leguminosae | *Cassia siamea* Lam. |  |  | 0.9 | 0.6 |  |
|  | *Leucaena leucocephala* (Lam.) de Wit |  |  | 0.2 | 47.7 |  |
|  | *Pithecellobium dulce* (Roxb.) Benth. |  |  |  | 1.7 |  |
|  | *Tamarindus indica* L. | 2.0 |  | 0.8 | 154.7 |  |
| Myrtaceae | *Psidium guajava* L. | 0.5 |  | 0.9 |  |  |

**Appendix 2.** Wild food plants observed in the dry season, classified by growth form and ordered by family and scientific name, indicating frequency of occurrence (Freq_SS_) as percentage of sampling sites where a species occurred in a spatial configuration.

| Botanical family | Scientific name | Fenced plot | Fenced plot margin | Yard | Home garden boundary | Pot |
| --- | --- | --- | --- | --- | --- | --- |
| Climber |  |  |  |  |  |  |
| Cucurbitaceae | *Coccinia grandis* (L.) Voigt | 0.17 | 0.08 | 0.15 |  |  |
|  | *Momordica charantia* L. |  |  | 0.15 | 0.08 | 0.05 |
| Menispermaceae | *Cissampelos pareira* L. | 0.08 | 0.08 | 0.15 |  |  |
|  | *Tiliacora triandra* Diels | 0.08 |  | 0.20 | 0.17 | 0.05 |
| Rattan |  |  |  |  |  |  |
| Arecaceae | *Calamus* sp. |  |  | 0.20 |  |  |
| Shrub |  |  |  |  |  |  |
| Leguminosae | *Cajanus cajan* (L.) Millsp. |  |  | 0.05 |  |  |
|  | *Senna sophera* (L.) Roxb. |  |  | 0.05 |  |  |
| Terrestrial herb |  |  |  |  |  |  |
| Amaranthaceae | *Amaranthus viridis* L. | 0.08 | 0.08 | 0.05 |  |  |
| Convolvulaceae | *Ipomoea aquatica* Forssk. |  |  | 0.10 |  |  |
| Umbelliferae | *Centella asiatica* (L.) Urb. | 0.08 |  | 0.05 |  | 0.10 |
| Tree |  |  |  |  |  |  |
| Anacardiaceae | *Spondias pinnata* Kurz | 0.08 |  | 0.05 | 0.08 |  |
| Clusiaceae | *Cratoxylum formosum* (Jack) Benth. & Hook.f. ex Dyer | 0.08 |  | 0.10 |  |  |
| Euphorbiaceae | *Phyllanthus acidus* (L.) Skeels | 0.08 |  | 0.40 | 0.17 |  |
| Lecythidaceae | *Barringtonia acutangula* (L.) Gaertn. |  |  | 0.15 |  |  |
| Leguminosae | *Cassia siamea* Lam. |  |  | 0.10 | 0.08 |  |
|  | *Leucaena leucocephala* (Lam.) de Wit |  |  | 0.15 | 0.58 |  |
|  | *Pithecellobium dulce* (Roxb.) Benth. |  |  |  | 0.17 |  |
|  | *Tamarindus indica* L. | 0.17 |  | 0.50 | 0.50 |  |
| Myrtaceae | *Psidium guajava* L. | 0.08 |  | 0.10 |  |  |

**Appendix 3.** Wild food plants observed in the rainy season, classified by growth form and ordered by family and scientific name, indicating absolute abundance (Ab) as number of individuals of a species per 100 m^2^.

| Botanical family | Scientific name | Fenced plot | Fenced plot margin | Yard | Home garden boundary | Pot |
| --- | --- | --- | --- | --- | --- | --- |
| Climber |  |  |  |  |  |  |
| Cucurbitaceae | *Coccinia grandis* (L.) Voigt |  |  | 0.9 |  |  |
|  | *Momordica charantia* L. | 0.5 | 0.8 | 0.4 |  |  |
| Menispermaceae | *Cissampelos pareira* L. | 2.0 |  | 0.4 |  |  |
|  | *Tiliacora triandra* Diels |  |  | 0.3 |  | 33.3 |
| Rattan |  |  |  |  |  |  |
| Arecaceae | *Calamus* sp. |  |  | 0.3 |  |  |
| Shrub |  |  |  |  |  |  |
| Leguminosae | *Cajanus cajan* (L.) Millsp. |  | 2.4 |  |  |  |
|  | *Senna sophera* (L.) Roxb. |  |  | 0.4 |  |  |
| Terrestrial herb |  |  |  |  |  |  |
| Amaranthaceae | *Amaranthus viridis* L. | 2.2 | 2.4 | 0.6 |  |  |
| Convolvulaceae | *Ipomoea aquatica* Forssk. |  | 1.6 | 0.4 |  |  |
| Scrophulariaceae | *Limnophila aromatica* Merr. |  |  |  |  | 2633.3 |
| Umbelliferae | *Centella asiatica* (L.) Urb. |  |  |  |  | 433.3 |
| Tree |  |  |  |  |  |  |
| Anacardiaceae | *Spondias pinnata* Kurz | 0.5 | 0.8 | 0.9 | 0.6 |  |
| Clusiaceae | *Cratoxylum formosum* (Jack) Benth. & Hook.f. ex Dyer | 0.5 |  | 0.9 |  |  |
| Euphorbiaceae | *Phyllanthus acidus* (L.) Skeels |  |  | 0.4 | 1.7 |  |
| Lecythidaceae | *Barringtonia acutangula* (L.) Gaertn. |  |  | 0.1 |  |  |
| Leguminosae | *Cassia siamea* Lam. |  |  | 0.2 |  |  |
|  | *Leucaena leucocephala* (Lam.) de Wit |  |  |  | 31.4 |  |
|  | *Pithecellobium dulce* (Roxb.) Benth. |  |  |  | 0.6 |  |
|  | *Tamarindus indica* L. |  |  | 0.8 | 215.1 |  |
| Myrtaceae | *Psidium guajava* L. |  |  | 0.9 |  |  |

**Appendix 4.** Wild food plants observed in the rainy season, classified by growth form and ordered by family and scientific name, indicating frequency of occurrence (Freq_SS_) as percentage of sampling sites where a species occurred in a spatial configuration.

| Botanical family | Scientific name | Fenced plot | Fenced plot margin | Yard | Home garden boundary | Pot |
| --- | --- | --- | --- | --- | --- | --- |
| Climber |  |  |  |  |  |  |
| Cucurbitaceae | *Coccinia grandis* (L.) Voigt |  |  | 0.10 |  |  |
|  | *Momordica charantia* L. | 0.08 | 0.08 | 0.05 |  |  |
| Menispermaceae | *Cissampelos pareira* L. | 0.17 |  | 0.05 |  |  |
|  | *Tiliacora triandra* Diels |  |  | 0.30 |  | 0.05 |
| Rattan |  |  |  |  |  |  |
| Arecaceae | *Calamus* sp. |  |  | 0.15 |  |  |
| Shrub |  |  |  |  |  |  |
| Leguminosae | *Cajanus cajan* (L.) Millsp. |  | 0.08 |  |  |  |
|  | *Senna sophera* (L.) Roxb. |  |  | 0.05 |  |  |
| Terrestrial herb |  |  |  |  |  |  |
| Amaranthaceae | *Amaranthus viridis* L. | 0.08 | 0.08 | 0.10 |  |  |
| Convolvulaceae | *Ipomoea aquatica* Forssk. |  | 0.08 | 0.10 |  |  |
| Scrophulariaceae | *Limnophila aromatica* Merr. |  |  |  |  | 0.19 |
| Umbelliferae | *Centella asiatica* (L.) Urb. |  |  |  |  | 0.05 |
| Tree |  |  |  |  |  |  |
| Anacardiaceae | *Spondias pinnata* Kurz | 0.08 | 0.08 | 0.10 | 0.08 |  |
| Clusiaceae | *Cratoxylum formosum* (Jack) Benth. & Hook.f. ex Dyer | 0.08 |  | 0.10 |  |  |
| Euphorbiaceae | *Phyllanthus acidus* (L.) Skeels |  |  | 0.25 | 0.08 |  |
| Lecythidaceae | *Barringtonia acutangula* (L.) Gaertn. |  |  | 0.15 |  |  |
| Leguminosae | *Cassia siamea* Lam. |  |  | 0.20 |  |  |
|  | *Leucaena leucocephala* (Lam.) de Wit |  |  |  | 0.50 |  |
|  | *Pithecellobium dulce* (Roxb.) Benth. |  |  |  | 0.08 |  |
|  | *Tamarindus indica* L. |  |  | 0.50 | 0.50 |  |
| Myrtaceae | *Psidium guajava* L. |  |  | 0.10 |  |  |
